# New sporopollenin-based β-cyclodextrin functionalized magnetic hybrid adsorbent for magnetic solid-phase extraction of nonsteroidal anti-inflammatory drugs from water samples

**DOI:** 10.1098/rsos.171311

**Published:** 2018-07-18

**Authors:** Syed Fariq Fathullah Syed Yaacob, Muhammad Afzal Kamboh, Wan Aini Wan Ibrahim, Sharifah Mohamad

**Affiliations:** 1 Department of Chemistry, Faculty of Science, University of Malaya, Kuala Lumpur, Malaysia; 2 University Malaya Centre for Ionic Liquids (UMCiL), University of Malaya, Kuala Lumpur, Malaysia; 3 Department of Chemistry, Shaheed Benazir Bhutto University, Shaheed Benazirabad, Sindh, Pakistan; 4 Separation Science and Technology Group (SepSTec), Department of Chemistry, Faculty of Science, Universiti Teknologi Malaysia, Johor Bahru, Johor, Malaysia

**Keywords:** sporopollenin, magnetic nanoparticles, β-cyclodextrin, NSAIDs, magnetic solid-phase extraction

## Abstract

A magnetic solid-phase extraction (MSPE) procedure on the newly synthesized magnetic β-cyclodextrin functionalized with toluene diisocyanate (TDI) as a linker and further modified with bio-polymeric spores of sporopollenin (MSp-TDI-βCD), was developed for the extraction of nonsteroidal anti-inflammatory drugs (NSAIDs), namely, indoprofen (INP), ketoprofen (KTP), ibuprofen (IBP) and fenoprofen (FNP) from water samples prior to their HPLC-DAD determination. The newly synthesized MSp-TDI-βCD was comprehensibly characterized using FT-IR, XRD, SEM-EDX, BET and VSM analyses. The separation of selected NSAIDs on MSp-TDI-βCD from aqueous solution was simply achieved by applying an external magnetic field via a permanent magnet. The MSPE parameters affecting extraction performance, i.e. sorbent dosage, sample volume, extraction and desorption time, type of organic eluent and volume and solution pH were investigated and optimized. The proposed method showed linear range between 0.5 and 500 ng ml^−1^, low limit of detection at S/N = 3 (0.16–0.37 ng ml^−1^) and limit of quantification at S/N = 10 (0.53–1.22 ng ml^−1^). The inter-day (*n =* 15) and intra-day (*n =* 5) precision for the proposed methods given by relative standard deviation (RSD%) was in the range of 2.5–4.0 and 2.1–5.5, respectively. The extraction recoveries of NSAIDs from environmental samples (tap, drinking and river water) ranged from 92.5% to 123.6%, with satisfactory precision (RSD% less than 12.4%).

## 1. Introduction

In the past few decades, massive manufacturing of pharmaceutical products has been creating serious environmental problems because contaminated effluents of pharmaceutical products contain highly stable and persistent environmental pollutants [1–3]. Numerous non-metabolized drugs which are not fully absorbed by and are excreted from the human body generally may reach the waste compartment through effluents [4]. Wastewater from hospitals, pharmaceutical industries and private households, by virtue of these therapeutic drug agents, leads to the contamination of water sources and poses a severe threat to human health [1,2]. Undoubtedly, nonsteroidal anti-inflammatory drugs (NSAIDs) are renowned as one of the most subscribed drugs worldwide and are used to relieve inflammatory, chronic and acute pain conditions such as osteoarthritis and orthopaedic fracture. In addition, NSAIDs also possess an antipyretic as well as analgesic property that has been used in human and veterinary medication. But, due to poor degradation factor and high water solubility, prolonged consumption of these drugs leads to extensive water contamination. Moreover, according to the international Council Directive 96/23/EC report, among the pharmaceutical products, NSAIDs have been recognized as perilous residues [4]. Consequently, taking into consideration the detrimental effects, the US Food and Drug Administration (FDA) established a maximum allowed concentration of pharmaceutical compound of 1.0 ng ml^−1^ into aquatic environment [3]. Therefore, by virtue of well-known toxicity and detrimental effect, the precise determination and detoxification of NSAIDs contaminated aqueous environment is of significant importance [4,5]. Literature survey indicated that determination of NSAIDs in waste water samples, involving pre-concentration step and separation followed by quantification, is necessary due to its low concentration in complex matrix [6,7]. More than that, the selectivity issues also play an important role in the determination of NSAIDs in water samples [8].

Several techniques, including liquid–liquid extraction (LLE) [9], liquid-phase microextraction (LPME) [10], solid-phase microextraction (SPME) [11] and solid-phase extraction (SPE) [12], have been applied to remediate the NSAIDs and their degraded products from the aqueous environment. Majority of these techniques are tedious and time-consuming with consequent risks such as use of toxic solvents in LLE and use of expensive cartridges in SPE [13–15]. SPME also encounters limitation related to low extraction efficiencies and low reproducibility and robustness because of fragile fibres as well as use of expendable solid-phase cartridges which have limited lifetime [16,17]. Comparatively, magnetic solid-phase extraction (MSPE), due to its simplicity, significant recovery, short extraction time, high enrichment factor and low cost, is renowned as an advantageous and authentic method [18]. Abundant literature reported that MSPE has been comprehensibly used to intoxicate the phenol [19], PAHs [20] and [21] contaminated effluents of pesticides [22–24]. As MSPE is based on magnetic sorbent, the exploitation of innovative highly selective NSAID sorbents, which can be easily regenerated and effectively used in aqueous media, has currently become a focus of intensive research [6,25]. Fe_3_O_4_ magnetic nanoparticles (MNPs), due to large surface area, extraordinary paramagnetic properties, stability, less toxicity, ease of synthesis as well as versatile functionalization, deserve particular attention [6,26]. Renowned bio-polymeric material, i.e. sporopollenin, is recognized as a potential SPE biosorbent by virtue of its remarkable physical, chemical/biological stability and chemical reactivity. Additionally, the exines are hollow, 2 mm thick walls perforated with numerous channels which make them porous so that both the outer and inner surfaces are potentially available for binding/magnetization [27].

**Figure 1.**
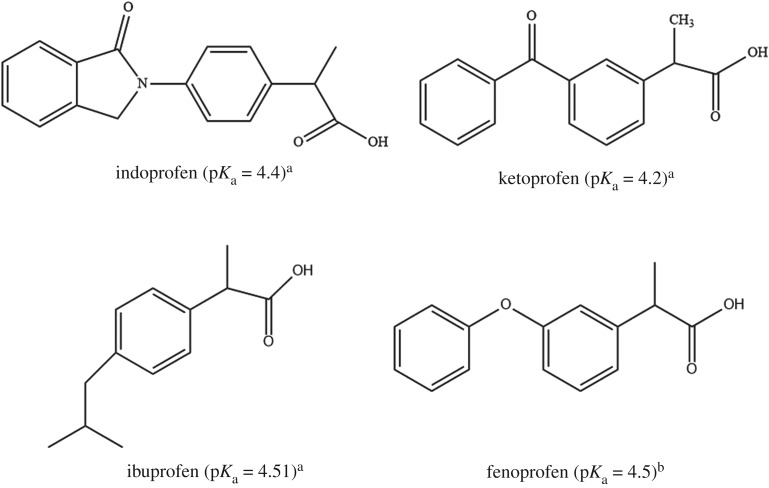
The chemical structures of the selected NSAIDs and p*K*_a_ value. ^a^De Oliveira *et al.* [11]. ^b^See http://www.drugbank.ca (accessed on 6 July 2017).

On the other hand, the second generation of synthetic supramolecules, i.e. cyclodextrins (CDs), has received considerable importance in the field of host–guest chemistry due to its tendency to bind the variety of pollutants with unique size-selectivity [22,28,29]. CDs are torus-shaped cyclic oligosaccharides with an internal hydrophobic cavity containing 6–12 glucose units joined together by α-1,4-glycosidic linkages [30]. In the last three decades, CD-based materials have been significantly developed owing to CDs’ unique character which can form noncovalent inclusion complex with other compounds through host–guest interactions by encapsulating either completely or partially fit into the lipophilic cavity [31]. The cavity of CDs provides a hydrophobic space in which a guest can be sequestered in an aqueous medium. CDs are known to form stable complexes with a wide range of compounds, including dyes, organic compounds and metal ions [32]. To the best of our knowledge, there is no example of βCD functionalized toluene diisocyanate (TDI)-modified bio-polymeric spores of sporopollenin (MSp-TDI-βCD)-based MSPE sorbent for the extraction of NSAIDs [26]. Consequently, in the current study we intend to develop a green MSPE sorbent by using bio-polymer material and supramolecular host molecule, βCD which is grafted onto sporopollenin. The βCD-functionalized TDI-modified bio-polymeric spores were magnetized with iron oxide nanoparticles (MSp-TDI-βCD). The synthesized MSp-TDI-βCD was successfully employed as an MSPE sorbent for the extraction of selected NSAIDs (figure 1) from water samples prior to their HPLC-DAD determination.

## 2. Material and methods

### 2.1. Materials

‘*Lycopodium clavatum*’ sporopollenin with a particle size of 25 µm was purchased from Aldrich (Steinheim, Germany). All chemicals used were of analytical grade and purchased from Merck (Darmstadt, Germany) or Sigma (Steinheim, Germany) and used without further purification. All commercial grade solvents stored over molecular sieves (4 Å, 8–12 mesh) from (Steinheim, Germany). The pH of the solution was adjusted by mixing appropriate amount of HCl and/or NaOH (0.1 M). Deionized water that had been passed through a Milli-Q system (Lane End, UK) was used for the preparation of solutions.

### 2.2. Instruments

The FT-IR spectrum was obtained using ATR mode on a Spectrum 400 Perkin Elmer in the range of 4000–450 cm^−1^ with diamond as a detector. The SEM-EDX analysis was performed using HITACHI SU8220 scanning electronic microscopy from OXFORD Instrument (Oxfordshire, UK). The magnetization of functionalized MNP was measured using vibrating sample magnetometer (VSM LakeShore 7400 series). X-ray diffraction (XRD) patterns of the samples were taken using PANalytical Empyrean X-ray diffractometer (EA Almelo, The Netherlands) from 2Θ = 15° to 75° at room temperature using Cu K_α_ radiation at a wavelength of 1.5418 Å at a scan rate of 0.02 s^−1^. The surface area and porosity were measured using Brunauer–Emmett–Teller (BET) by nitrogen adsorption–desorption isotherm in Micromeritics Tristar II ASAP 2020, (GA, USA). HPLC system (Kyoto, Japan), consisting of LC-20AT pump, SPD-M20A diode array detector, SIL-20A HT autosampler and CTO-10AS VP column oven, was used for NSAID determination. The system was equipped with a Hypersil gold C-18 reverse phase column (250 × 4.6 mm), particle size (5 µm) from ThermoScience (MA, USA).

### 2.3. HPLC parameters and conditions

HPLC-DAD system was used for the chromatographic identification of the selected NSAIDs from the water samples. The chromatographic separation was carried out using acidified (1% with acetic acid) water/acetonitrile (50 : 50 v/v) as a mobile phase at a flow rate of 1 ml min^−1^ for selected NSAIDs. The HPLC column temperature was set at 40°C. The sample inject volume was 10 µl. The DAD detection for the selected NSAIDs was carried out at multiple wavelengths, i.e. 281, 255, 271 and 219 nm for indoprofen (INP), ketoprofen (KTP), ibuprofen (IBP) and fenoprofen (FNP) respectively.

### 2.4. Synthesis

#### 2.4.1. Functionalization of sporopollenin with TDI (Sp-TDI)

Two grams of sporopollenin and 10 ml of TDI were mixed using a magnetic stirrer in 20 ml dry toluene. The reaction mixture was stirred for 4 h at room temperature under inert atmosphere. Then, the resultant Sp-TDI was separated by centrifugation at 1792 RCF for 5 min and sequentially washed with excess of dry toluene. The sample was dried and stored in a desiccator.

#### 2.4.2. Preparation of βCD-based TDI-modified sporopollenin (Sp-TDI-βCD)

In 20 ml of dry N,N-dimethylformamide (DMF), 0.7 g of βCD was dissolved, following the addition of 1.5 g of freshly prepared Sp-TDI; the reaction mixture was stirred under nitrogen atmosphere at 70°C for 2 h. Then the resultant Sp-TDI-βCD was washed with excess of acetone and deionized water for the removal of unreacted particles and dried under vacuum at 60°C.

#### 2.4.3. Preparation of magnetic βCD-based sporopollenin (MSp-TDI-βCD)

**Figure 2.**
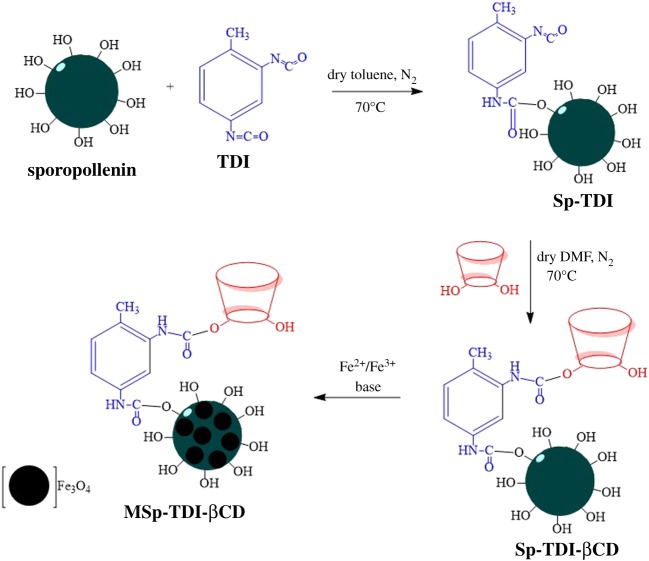
Schematic routes for the synthesis of MSp-TDI-βCD sorbent.

The magnetization of βCD-functionalized TDI-modified sporopollenin (Sp-TDI-βCD) was carried out as follows: 13.32 g of FeCl_3_.6H_2_O, 19.88 g of FeCl_2_.4H_2_O, 5 ml of HCl (5 M), 40 ml of deionized water and 5 ml of ethanol were mixed in a flask followed by heating to 40°C until complete dissolution of the salts. Then 1.0 g of the freshly prepared Sp-TDI-βCD was redispersed in 30 ml of the solution and stirred for 2 h at room temperature. The Sp-TDI-βCD suspension was filtered and the filtrate was quickly washed with deionized water and immediately transferred to a 0.1 M ammonia solution. After 2 h stirring at room temperature, the ‘magnetic βCD-functionalized TDI-modified sporopollenin’ (MSp-TDI-βCD) was separated from the solution using an external magnet and washed thoroughly with deionized water and dried under vacuum. Figure 2 illustrates the synthesis pathway of MSp-TDI-βCD sorbent.

### 2.5. Magnetic solid-phase extraction

For MSPE of NSAIDs, 10 mg MSp-TDI-βCD sorbent was added to 200 ml of deionized water spiked with the mixture of INP, KTP, IBP and FNP with pH 4. The mixture was stirred vigorously for 30 min to make the sorbent disperse uniformly in the solution. Then, the sorbent was isolated from the solution by using an external magnet. The solution became limpid and the upper solution was decanted after 5 min. Then, 1.5 ml of acetonitrile was added to elute the NSAIDs adsorbed on MSp-TDI-βCD and was horizontally shaken for 30 min. The collected eluate was dried using stream nitrogen gas and re-dissolved in 0.7 ml of acetonitrile. Finally, 10 µl portion of the eluate was injected into HPLC for analysis. Furthermore, enrichment factor can be calculated which is the ratio of highest sample volume over lowest sample volume [33–35]. In the current study, the highest volume and the lowest sample volume were 200 ml and 0.7 ml, respectively.

### 2.6. Real sample preparation

Three environmental water samples, namely tap, drinking and river water were used to assess the field application of the synthesized MSp-TDI-βCD towards the extraction of the selected NSAIDs. Tap water sample was obtained from the analytical chemistry laboratory of University of Malaya, Malaysia, while river and drinking water samples were collected from Sungai Sendat, Selangor, Malaysia. The collected samples were stored at 4°C prior to use.

## 3. Results and discussion

### 3.1. Synthesis

The aim of this study was to design a new bio-polymer hybrid sporopollenin-based magnetic material with supramolecular βCD host and investigate its extraction properties towards the selected NSAIDs. In the first step, sporopollenin surface was connected with TDI as a linker by connecting at para position of isocyanate group. In the second step, the sporopollenin surface attached with TDI was functionalized with βCD after inter-connecting at ortho position of isocyanate in the presence of dry DMF. In the final step, in order to adopt magnetic behaviour sorbent, magnetization of sporopollenin surface was achieved by the embedded MNPs inside the pores of Sp-TDI-βCD surface by adding Fe^2+^ and Fe^3+^ ions in the presence of basic condition. Finally, the resultant bio-polymer hybrid MSp-TDI-βCD sorbent was chosen as the precursor.

### 3.2. Characterization of sorbent

Functionalization of sporopollenin with TDI (synthesis of Sp-TDI), modification of βCD with TDI functionalized sporopollenin (synthesis of Sp-TDI-βCD) and magnetization of Sp-TDI-βCD (synthesis of MSp-TDI-βCD) were comprehensibly confirmed by using FT-IR, SEM-EDX spectroscopy as well as XRD, BET and VSM analysis.

**Figure 3.**
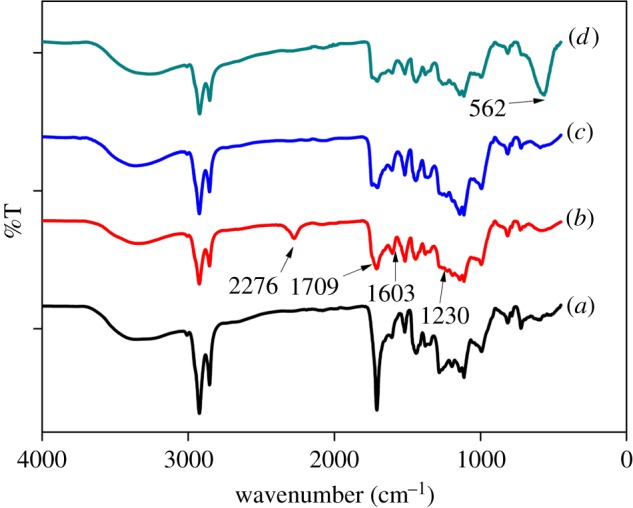
FTIR spectra of (*a*) sporopollenin, (*b*) Sp-TDI, (*c*) Sp-TDI-βCD and (*d*) MSp-TDI-βCD.

Sporopollenin following the functionalization process with TDI shows some additional bands at around 2276, 1709, 1603 and 1230 cm^−1^ for N=C=O, C=O and C–N groups (figure 3*b*). The appearance of isocyanate and carbonyl bands at 2276, 1709 and 1603 cm^−1^ clearly indicates the incorporation of isocyanate functionalities on the surface of sporopollenin. Additional peak at 1230 cm^−1^ (figure 3*b*) for C–N groups stretching also confirmed the successful functionalization. On the other hand, disappearance of characteristic peaks of isocyanate group (N=C=O) vibration shown in figure 3*c* at 2276 cm^−1^ due to the reaction between isocyano group with βCD is qualitative evidence for the formation of Sp-TDI-βCD. Hence, it indicates that βCD is successfully bonded onto the surface of the Sp-TDI. Moreover, all significant peaks of βCD in the range of 900–1709 cm^−1^ were present with a small shift. Both TDI-grafted sporopollenin (Sp-TDI) and βCD-modified sporopollenin (Sp-TDI-βCD) have very similar absorption patterns, but the FT-IR spectral analysis shows clearly different bands at around 562 cm^−1^. The magnetization of Sp-TDI-βCD with Fe_3_O_4_ MNPs and formation of MSp-TDI-βCD can be confirmed by the appearance of Fe-O stretching at around 562 cm^−1^ (figure 3*d*).

**Figure 4.**
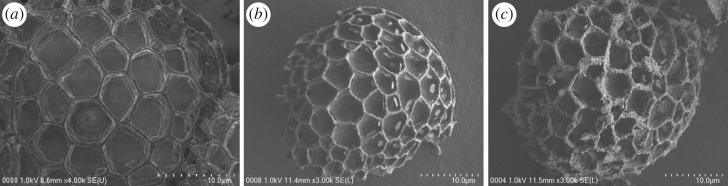
SEM images of (*a*) sporopollenin, (*b*) Sp-TDI-βCD and (*c*) MSp-TDI-βCD.

**Figure 5.**
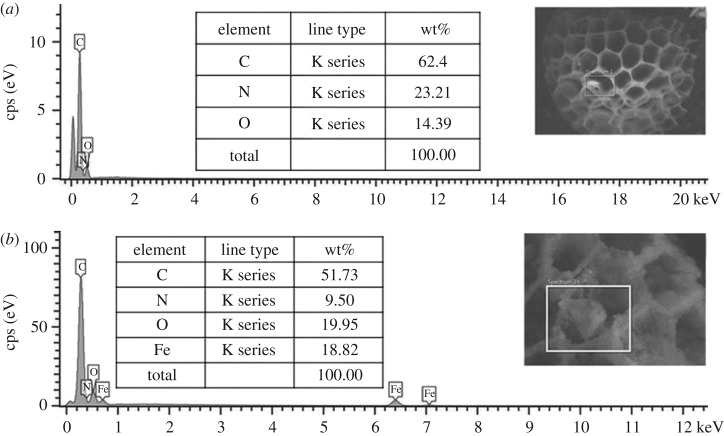
EDX spectra of (*a*) Sp-TDI-βCD and (*b*) MSp-TDI-βCD.

The morphological and structural analysis of the synthesized Sp-TDI-βCD and MSp-TDI-βCD were studied through scanning electron microscope (SEM). From the SEM micrograph of the raw sporopollenin (figure 4*a*), a smooth morphology of the sporopollenin network with open and uniform interconnected pore structure can be seen. While the SEM image of Sp-TDI-βCD (figure 4*b*) showed that following the modification process with βCD the pores of sporopollenin changed to be rough and bumpy filled with βCD molecules. SEM micrograph of the MSp-TDI-βCD (figure 4*c*) reveals that the magnetite nanoparticles are predominantly localized inside the open pores of sporopollenin. Hence, these images reveal that the magnetic nanoparticles have been embedded inside the pores of sporopollenin. Moreover, to confirm the formation of Sp-TDI-βCD and MSp-TDI-βCD and to investigate the elemental composition as well as distribution of magnetic particles in the newly prepared sorbents, energy-dispersive X-ray spectroscopy (EDX) was performed using the SEM-EDX analysis. The obtained EDX spectrum of Sp-TDI-βCD along with SEM image (figure 5*a*) obviously reveals the formation of Sp-TDI-βCD. Raw sporopollenin only contains hydrogen, carbon and oxygen [36]. EDX results (figure 5*a*) showed that 23.21% of nitrogen originated from isocyanate group after functionalization of sporopollenin with TDI molecule. Meanwhile, after magnetization process with Fe_3_O_4_ EDX spectrum of MSp-TDI-βCD (figure 5*b*) showed the presence of 18.82% of iron. The presence of iron magnetite indicates that the final material MSp-TDI-βCD was successfully filled with magnetic nanoparticle Fe_3_O_4_.

The XRD analyses were performed to measure the crystallinity of Sp-TDI, Sp-TDI-βCD and MSp-TDI-βCD. Sp-TDI exhibits broad diffraction peaks at approximately 25° which is typically observed for amorphous material (electronic supplementary material, figure S1). Meanwhile, few diffraction peaks appear at approximately 10°–20° when βCD was incorporated on the surface of Sp-TDI as βCD existed as crystalline form. Hence, it indicates that βCD improves the crystallinity of Sp-TDI-βCD (electronic supplementary material, figure S1*b*). According to Joint Committee on Powder Diffraction Standards (JCPDS) reference pattern of magnetite (00-019-0629), six diffraction peaks of Fe_3_O_4_ are observed in MSp-TDI-βCD in electronic supplementary material, figure S1*c*, which are (220), (311), (400), (422), (511), and (440) related to cubic spine plane of Fe_3_O_4_ and confirming the presence of Fe_3_O_4_. It is also observed that no distinct diffraction peaks of βCD appear for MSp-TDI-βCD, which indicates that the βCD molecules are distributed homogeneously without forming any phase-separated crystal aggregates [31,37].

The magnetism behaviour of MSp-TDI-βCD was characterized by the vibrating sample magnetometer (VSM) technique. MSp-TDI-βCD sample displayed typical superparamagnetic behaviour (electronic supplementary material, figure S2). The saturation magnetization of analysed sample was 31.49 emu g^−1^ and sufficient for magnetic separation as minimal magnetic separation reported was 16.30 emu g^−1^ [38]. The inset photos in electronic supplementary material, figure S2 showed the sorbent separation by using external magnetic field.

BET surface area measurements were also made on the MSp-TDI-βCD. Electronic supplementary material, figure S3 shows N_2_ adsorption–desorption which is close to type IV of the IUPAC classification with an evident hysteresis loop in the 0.05–1.0 range, suggesting that the sample is basically mesoporous [39]. The relatively high specific surface area of the sample calculated by BET is 16.5083 m^2^ g^−1^ and is related to the nanometric size of its particles. The surface area can be used to estimate the pore size according to the equation 4*V*/*S*_BET_, where *V* is adsorption total pore volume and *S*_BET_ is the specific surface area of the MSp-TDI-βCD. The pore size calculated from the surface area is approximately 22.3480 nm. Table 1 shows the pore size, pore volume and *S*_BET_ for sporopollenin, Sp-TDI-βCD and MSp-TDI-βCD. The escalation in the pore size can be caused by dispersity of particles that make surface area increased as well as increased pore volume. Thus, it will enhance the adsorption capacity especially for large adsorbate molecule [40].

**Table 1.** Physical properties of Sporopollenin, Sp-TDI-βCD and MSp-TDI-βCD.

sample	surface area (m^2^ g^−1^)	pore volume (cm^3^ g^−1^)	pore size (nm)
sporopollenin	2.2675	0.0014	2.5990
Sp-TDI-βCD	2.7263	0.0017	2.5091
MSp-TDI-βCD	16.5086	0.09223	22.348

### 3.3. Application of magnetic solid-phase extraction

In this study, INP, KTP, IBP and FNP were selected as target NSAID's drugs analyte in order to evaluate the MSPE extraction efficiency of the newly prepared MSp-TDI-βCD. The optimal condition was examined by evaluating various parameters including sorbent dosage, extraction time, sample volume, type of organic eluent, volume of organic eluent, desorption time and sample solution pH.

#### 3.3.1. Adsorbent dosage

**Figure 6.**
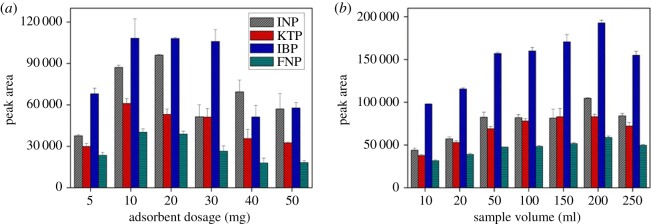
The effect of (*a*) adsorbent dosage, and (*b*) sample volume for the extraction of NSAIDs using MSp-TDI-βCD and analysis using HPLC-DAD. HPLC conditions: acidified (1% with acetic acid) water/acetonitrile (50 : 50 v/v) as a mobile phase at a flow rate of 1 ml min^−1^, the HPLC column temperature was set at 40°C, the sample injection volume was 10 µl, the DAD detection for the selected NSAIDs was carried out at multiple wavelengths, i.e. 281, 255, 271 and 219 nm for INP, KTP, IBP and FNP, respectively.

The effect of sorbent dosage on the per cent extraction of NSAIDs was assessed by changing the mass of sorbent (MSp-TDI-βCD) in the range of 5–50 mg (figure 6*a*). By increasing the amount of sorbent, peak area was increased significantly due to the accessible sites. However, the peak area was slightly decreased with further increasing of sorbent. This could be explained by weak elution efficiency of target analytes at specific sample volume and extraction/desorption time [41,42]. In addition, sorbent dosage has a different effect on the different target analytes due to the interaction between adsorption sites and the nature of NSAIDs. The quantitative extraction of four selected NSAIDs was obtained using 10 mg of the sorbent and it was sufficient to extract analytes from aqueous samples, thus selected for subsequent experiment.

#### 3.3.2. Sample volume

High enrichment factor can be obtained by using a large sample volume. Thus, the volume of water samples ranging from 20 to 250 ml was evaluated. From figure 6*b*, it can be ensured that highest peak area was obtained with 200 ml sample volume. However, beyond the 200 ml sample volume, the peak area decreased significantly probably due to the excessive breakthrough volume [12]. Therefore, the 200 ml sample volume was selected as the optimum volume for trace sample analysis of selected NSAIDs from water samples.

#### 3.3.3. Extraction and desorption time

**Figure 7.**
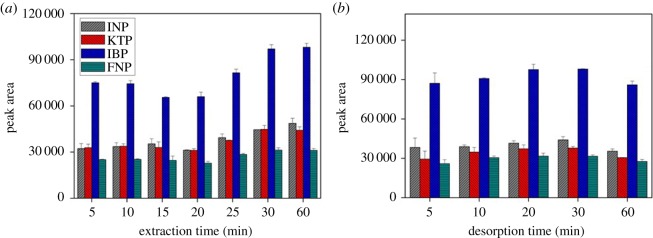
The effect of (*a*) extraction time, and (*b*) desorption time on the extraction of NSAIDs from 5 to 60 min using MSp-TDI-βCD and analysis using HPLC-DAD. HPLC conditions: acidified (1% with acetic acid) water/acetonitrile (50 : 50 v/v) as a mobile phase at a flow rate of 1 ml min^−1^, the HPLC column temperature was set at 40°C, the sample injection volume was 10 µl, the DAD detection for the selected NSAIDs was carried out at multiple wavelengths, i.e. 281, 255, 271 and 219 nm for INP, KTP, IBP and FNP, respectively.

The extraction and desorption time profiles were performed between 5 and 60 min (figure 7*a*,*b*). The maximum peak area was attained at 30 min for all selected NSAIDs and it remained almost at constant thereon. It can be concluded that extraction and desorption equilibrium between the aqueous phase and the sorbent was nearly reached after 30 min for both cases. Thus, 30 min extraction and desorption time were sufficient for the subsequent experiments.

#### 3.3.4. Type of organic eluent and volume

**Figure 8.**
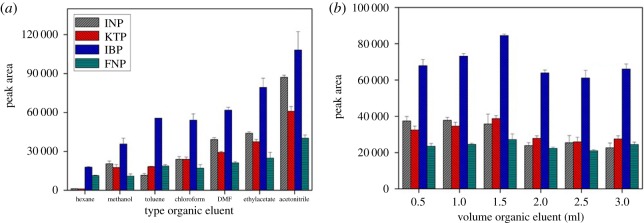
The effect of (*a*) type of organic eluent, and (*b*) volume of organic eluent for the extraction of NSAIDs using MSp-TDI-βCD and analysis using HPLC-DAD. HPLC conditions: acidified (1% with acetic acid) water/acetonitrile (50 : 50 v/v) as a mobile phase at a flow rate of 1 ml min^−1^, the HPLC column temperature was set at 40°C, the sample injection volume was 10 µl, the DAD detection for the selected NSAIDs was carried out at multiple wavelengths, i.e. 281, 255, 271 and 219 nm for INP, KTP, IBP and FNP, respectively.

Seven types of organic solvents, namely, methanol, acetonitrile, *n*-hexane, toluene, chloroform, ethyl acetate and N,N-dimethyl fluoride were used as desorption solvents to examine their effects on the extraction efficiencies of the selected NSAIDs. As shown in figure 8*a*, acetonitrile showed the best extraction of the selected NSAIDs with maximum peak area compared to other organic solvents. This phenomenon can be explained by the molecular interaction between analyte and sorbent surface. The predicted intermolecular forces that exist are hydrogen bonding, dipole–dipole interaction and also van der Waals forces. The role of organic eluent is to disrupt the retentive intermolecular forces between sorbent surface and analyte. Based on the result, it is expected that methanol gives strong eluent on polar polymeric sorbent, but for instance acetonitrile gives excellent eluent effect on sorbent surface compared with methanol. This is because methanol has strong polar eluent strength and it is incapable of disrupting non-polar interaction site. Acetonitrile is mid to polar–apolar eluent strength that can disrupt the binding mechanism at polar and non-polar site sorbent surface. *n*-hexane showed the poor performance eluent capability due to its non-polar characteristic which is incapable of disrupting the polar site in the presence of water adsorbent/absorbent on sorbent surface. The effect of the volume of the organic eluent was also determined. The volume ranging 0.5–3.0 ml of acetonitrile was optimized as shown in figure 8*b*. The maximum peak area was obtained with 1.5 ml of acetonitrile. Finally, 1.5 ml of acetonitrile was used in further experiment.

#### 3.3.5. Solution pH

**Figure 9.**
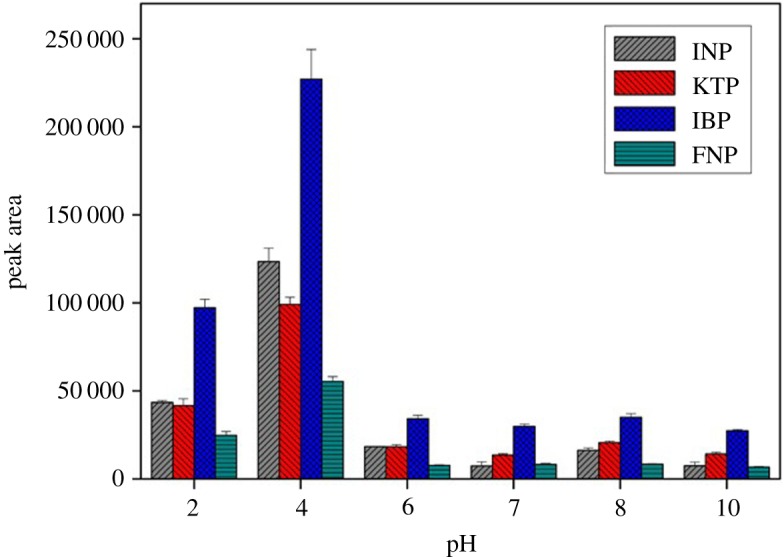
The effect of solution pH for the extraction of NSAIDs using MSp-TDI-βCD and analysis using HPLC-DAD. HPLC conditions: acidified (1% with acetic acid) water/acetonitrile (50 : 50 v/v) as a mobile phase at a flow rate of 1 ml min^−1^, the HPLC column temperature was set at 40°C, the sample injection volume was 10 µl, the DAD detection for the selected NSAIDs was carried out at multiple wavelengths, i.e. 281, 255, 271 and 219 nm for INP, KTP, IBP and FNP, respectively.

Among the different MSPE affecting parameters, the most important factor influencing the MSPE efficiency of a sorbent in wastewater treatment is the pH of the solution. The effectiveness of adsorption is highly dependent on the pH of the medium, as diversity in pH prompts variations in the surface properties of the sorbent and in the degree of ionization of the selected NSAID molecules. Consequently, the effect of solution pH on the extraction of selected NSAIDs was investigated using MSp-TDI-βCD as a sorbent at a pH range from 2 to 10. Results indicated that acidic medium is more suitable for the extraction of the selected NSAIDs and maximum efficiency for all the selected NSAIDs was obtained at pH 4. Beyond pH 4, peak area decreased dramatically. The highest peak area for the selected NSAIDs can be explained on the basis of p*K*_a_ values. The studied NSAID has p*K*_a_ value in the range of 4.0–4.5. As shown in electronic supplementary material, figure S4, the pH zero-point charge (pH_zpc_) for sorbent was determined at 6.0. At pH < p*K*_a_ value, the four NSAIDs mostly existed in deprotonated forms. The sorbent surface exists in protonated form, which resulted in electrostatic attraction between analytes and sorbent surface. Hence, higher extraction efficiency was obtained. At pH > 6, the extraction efficiency was low for targeted analytes due to electrostatic repulsion between NSAIDs and the sorbent surface. The NSAID molecule was transformed to anionic form, while the sorbent surface also changed to deprotonated surface. The extraction is affected in a negative way in which strong repulsive force occurred between the negative sorbent surface and anionic targeted analytes. Hence, the extraction is higher in acidic environment; pH 4.0 showed the maximum peak area, thus the value was selected for subsequent experiment (figure 9).

#### 3.3.6. Reusability of the sorbent

The stability and reusability of the sorbent are considered to be crucial factors for practical application and need to be systematically examined. Hence, reusability investigations of MSp-TDI-βCD were conducted to examine the effect of synthesized sorbent extraction efficiency after repeated usage cycles. After each cycle, the sorbent was washed with acetonitrile before the next MSPE application. The extraction efficiency remained stable after 5 recycles, which indicated that the MSp-TDI-βCD magnetic particles were mechanically stable and possessed good reusability.

### 3.4. Method validation

The MSp-TDI-βCD MSPE method was validated at optimized conditions: 10 mg sorbent dosage, 200 ml sample volume, 30 min extraction time, 30 min desorption time, 1.5 ml acetonitrile as organic eluent and sample solution pH 4. Under these optimal conditions, linearity, limit of detection (LOD), limit of quantification (LOQ) and precision (intra-day and inter-day) were evaluated to validate the developed method. As shown in table 2, all tested NSAIDs showed good linearity with good coefficiency of determination (*R*^2^ ≥ 0.990). The LOD was calculated based on signal-to-noise ratio (3 SD/*m*) (*n =* 10) and the values obtained were in the range 0.16–0.37 ng ml^−1^. The LOQ (10 × SD/*m*) (*n =* 10) was also calculated and the values obtained were in the range 0.53–1.22 ng ml^−1^. Precision was studied in terms of repeatability (intra-day) (*n =* 5) and reproducibility (inter-day) (*n =* 15) expressed as relative standard deviation (RSD). The inter-day precision was demonstrated by performing five replicates of standard solutions on a single day. The inter-day precision of the MSPE procedure was performed by performing standard solutions of the same concentration levels in five replicates on each of the three consecutive days. The linearity, LOD, LOQ and RSD% precision values are summarized in table 2.

**Table 2.** Qualitative data of the proposed MSPE method.

					precision	
analyte	linearity (ng ml^−1^)	*R*^2^	LOD (ng ml^−1^)	LOQ (ng ml^−1^)	intra (RSD% *n *= 5)	inter (RSD% *n *= 15)
INP	0.5–500	0.9944	0.16	0.53	4.7	2.7
KTP	0.5–500	0.9983	0.18	0.59	5.5	4.0
IBP	0.5–500	0.9927	0.37	1.22	2.1	2.5
FNP	0.5–500	0.9901	0.17	0.58	5.5	3.3

### 3.5. Environmental water sample analysis

**Figure 10.**
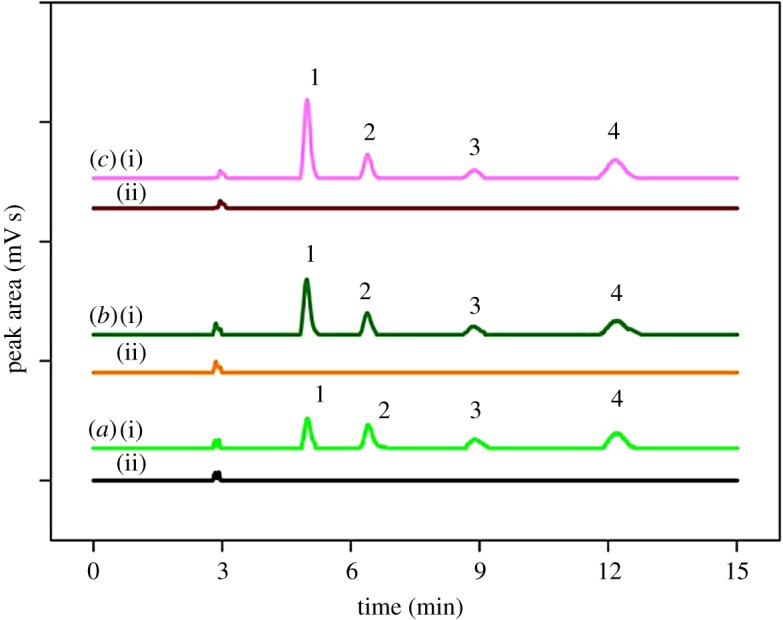
Chromatograms of NSAIDs (spiked at 10 ng ml^−1^ of each NSAIDs) using the proposed MSp-TDI-βCD MSPE method using spiked tap water sample (*a*)(i); non-spiked tap water sample (*a*)(ii); spiked drinking water sample (*b*)(i); non-spiked drinking water sample(*b*)(ii); spiked river water sample (*c*)(i); non-spiked river water sample (*c*)(ii). Peak identification; (1) INP (2) KTP (3) IBP and (4) FNP.

The performance of the developed MSp-TDI-βCD MSPE method for the extraction of selected NSAIDs was evaluated at optimum conditions for laboratory tap water, drinking water and river water samples to assess matrix effect on the three latter samples. For the field application, the real sample was spiked with 100 ng ml^−1^ and 10 ng ml^−1^ of NSAIDs. Based on the summarized results in table 3, the MSPE method gives better recovery of NSAIDs in tap water ranging 99.7–123.6% with RSDs (*n =* 5) in the range of 2.9–9.7%. For drinking water, the good recovery achieved ranging from 92.5% to 120.8% with RSDs (*n =* 5) from 1.9% to 12.3%. Meanwhile, the MSPE method also was tested in river water for the determination of NSAIDs and gives excellent recovery for spiked NSAIDs in the range of 97.3–123.5% with RSDs (*n =* 5) from 2.1% to 12.4%. Figure 10 shows the chromatogram of non-spiked and spiked 10 ng ml^−1^ of NSAIDs in three types of real samples.

**Table 3.** Percentage relative recovery and RSD (*n *= 5) for spiked NSAIDs from different water samples using MSp-TDI-βCD for the MSPE method.

		tap water (*n* = 5)		drinking water (*n* = 5)		river water (*n* = 5)	
analyte	spiked level (ng ml^−1^)	recovery (%)	RSD (%)	recovery (%)	RSD (%)	recovery (%)	RSD (%)
INP	10	123.6	9.5	92.9	4.1	112.8	4.2
	100	106.3	3.7	117.7	4.9	121.8	3.3
KTP	10	115.6	8.6	92.5	9.6	123.5	5.6
	100	112.8	4.0	103.5	8.4	97.3	3.4
IBP	10	109.4	8.2	113.7	12.3	103.4	9.4
	100	112.4	5.3	120.8	8.4	121.9	2.1
FNP	10	99.7	9.7	99.6	9.7	108.3	12.4
	100	118.5	2.9	110.3	1.9	98.9	7.7

### 3.6. Comparative study

Comparative analysis for the extraction of NSAIDs in terms of LOD, recovery and precision with current work is listed in table 4. The better results for LOD and recovery in comparison with reported sorbents showed that the newly fabricated MSp-TDI-βCD is more capable of extracting selected NSAIDs.

**Table 4.** Comparison of recovery and LOD of current work to other reported analytical technique and sample matrices.

matrix	type of sorbent	technique	recovery (%)	RSD (%)	LODs (ng ml^−1^)	ref.
seawater	molecularly imprinted polymers (MIPs)	MSPE/HPLC	86.3–103.5	2.12–4.33	4.5	[43]
water	magnetic graphene @polydopamine @Zr-MOF	MSPE/HPLC	64.8–92.8	0.62–4.89	0.1–1.0	[44]
human plasma	1-butyl-3-methylimidazolium bromide-INPs	MSPE/HPLC	98.7–100	0.4–0.8	1.5–5.8	[45]
aqueous sample	magnetic C_18_ microsphere	MSPE/GC	35.0–99.0	<10.0	0.8–36.0	[46]
milk sample	magnetic nanoparticles-graphene-cyanopropyltriethoxysilane (Fe_3_O_4_@G-CNPrTEOS)	MSPE/GC	82.0–104.0	4.1–9.1	0.1	[47]
tap, drinking and river water	MSp-TDI-βCD	MSPE/HPLC	92.5–123.6	1.9–12.4	0.1–0.4	current study

## 4. Conclusion

In this study, βCD-functionalized TDI-modified sporopollenin-based magnetic sorbent (MSp-TDI-βCD) was successfully synthesized and used as a new sorbent in MSPE for the simple, fast and efficient extraction of four selected NSAIDs from environmental water samples. Acquired results proved that the newly proposed MSPE method based on MSp-TDI-βCD was a rapid, reliable and highly efficient method for the extraction of NSAIDs namely INP, KTP, IBP and FNP from water sample with low LOD and RSD values with good per cent recoveries when compared with many reported materials. However, this developed method has limitation for quantification of IBP in aquatic environment due to the obtained LOQ value of IBP beyond the threshold limit. The field studies also supported the effectiveness of this new magnetic MSp-TDI-βCD sorbent which could be useful and has good potential for the extraction of selected NSAIDs from real water samples.

## Supplementary Material

Figures S1 - S4

## Data accessibility

All the datasets supporting this article are available at the Dryad Digital Repository (http://dx.doi.org/10.5061/dryad.hj2j3) [48].
