# Supplementary material for: New sporopollenin-based β-cyclodextrin functionalized magnetic hybrid adsorbent for magnetic solid-phase extraction of nonsteroidal anti-inflammatory drugs from water samples
Source: R Soc Open Sci. 2018 Jul 18;5(7):171311. doi: 10.1098/rsos.171311 (PMC6083704; doi:10.1098/rsos.171311)
Supplement: Figures S1 - S4 [file rsos171311supp1.docx]

**Electronic Supplementary Material Information of New Sporopollenin Based β-Cyclodextrin Functionalized Magnetic Hybrid Adsorbent for Magnetic Solid-Phase Extraction of NSAIDs from Water Samples**

Syed Fariq Fathullah Syed Yaacob^1^, Muhammad Afzal Kamboh^2^, Wan Aini Wan Ibrahim^3^, Sharifah Mohamad^1, 4*^

^1^Department of Chemistry, Faculty of Science, University of Malaya, Kuala Lumpur, Malaysia

^2^Department of Chemistry, Shaheed Benazir Bhutto University, Shaheed Benazirabad, Sindh, Pakistan

^3^Separation Science and Technology Group (SepSTec), Department of Chemistry, Faculty of Science, Universiti Teknologi Malaysia, Johor Bahru, Johor, Malaysia

^4^University Malaya Centre for Ionic Liquids (UMCiL), University of Malaya, Kuala Lumpur, Malaysia

*Corresponding author: [sharifahm@um.edu.my](mailto:sharifahm@um.edu.my)

Phone: +603-7967 6751; Fax: +60-379674193


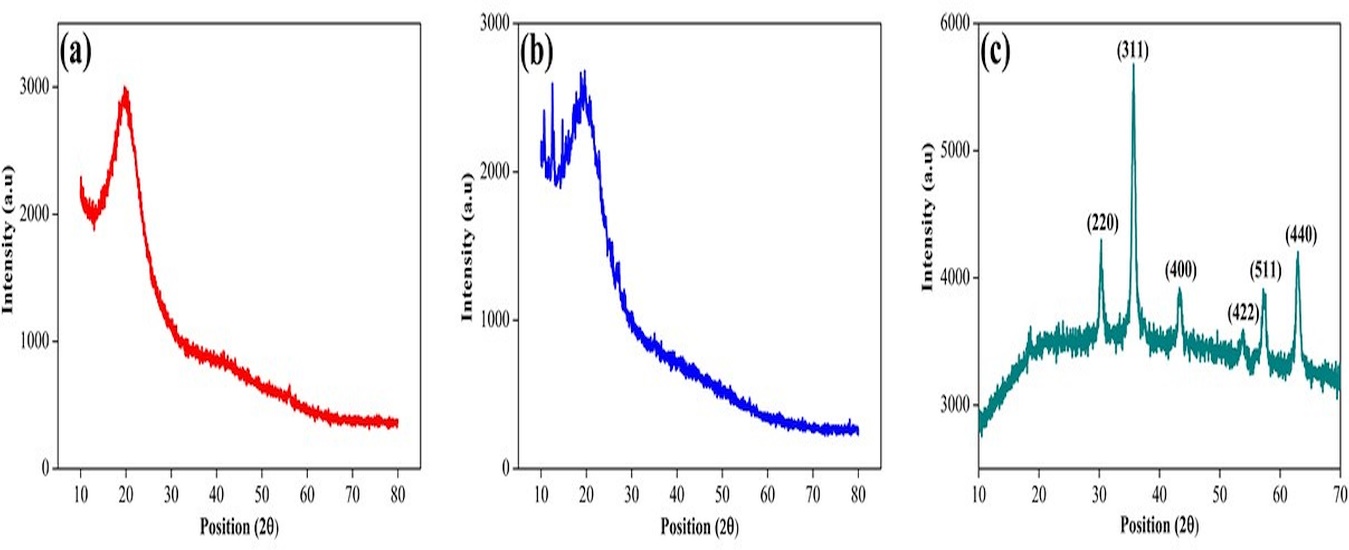


**Figure S1.** XRD pattern of (a) Sp-TDI, (b) Sp-TDI-βCD and (c) MSp-TDI-βCD

**
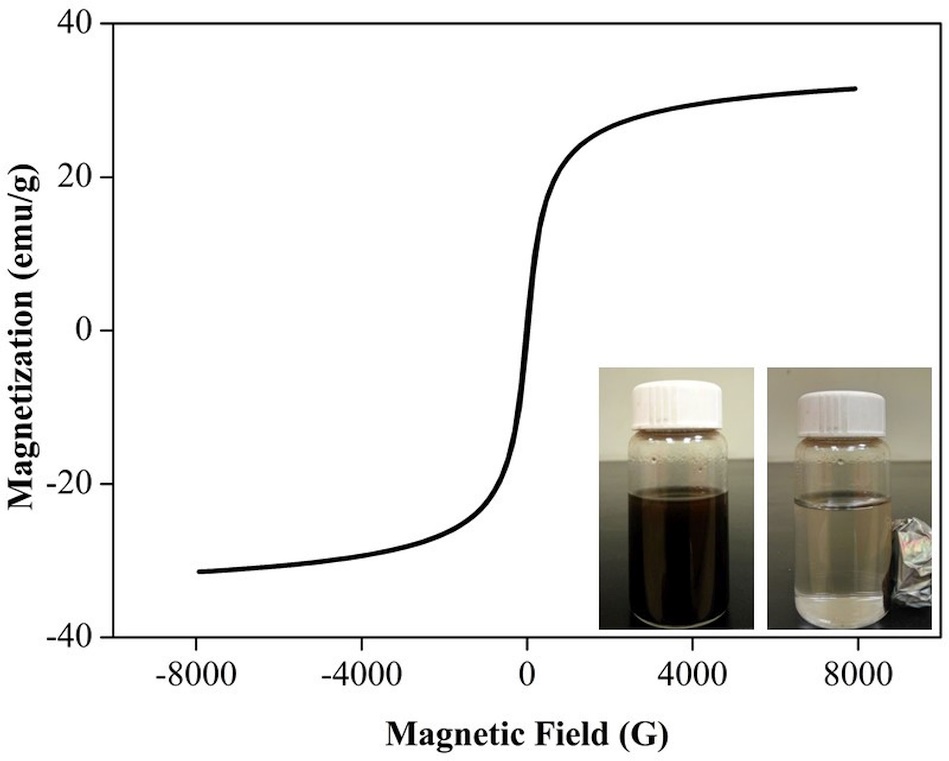
**

**Figure S2.** Magnetization curve of MSp-TDI-βCD. The inset shows photograph of magnetic biopolymer sorbent dispersed in aqueous solution (left) and separated from aqueous solution under an external magnetic field (right)


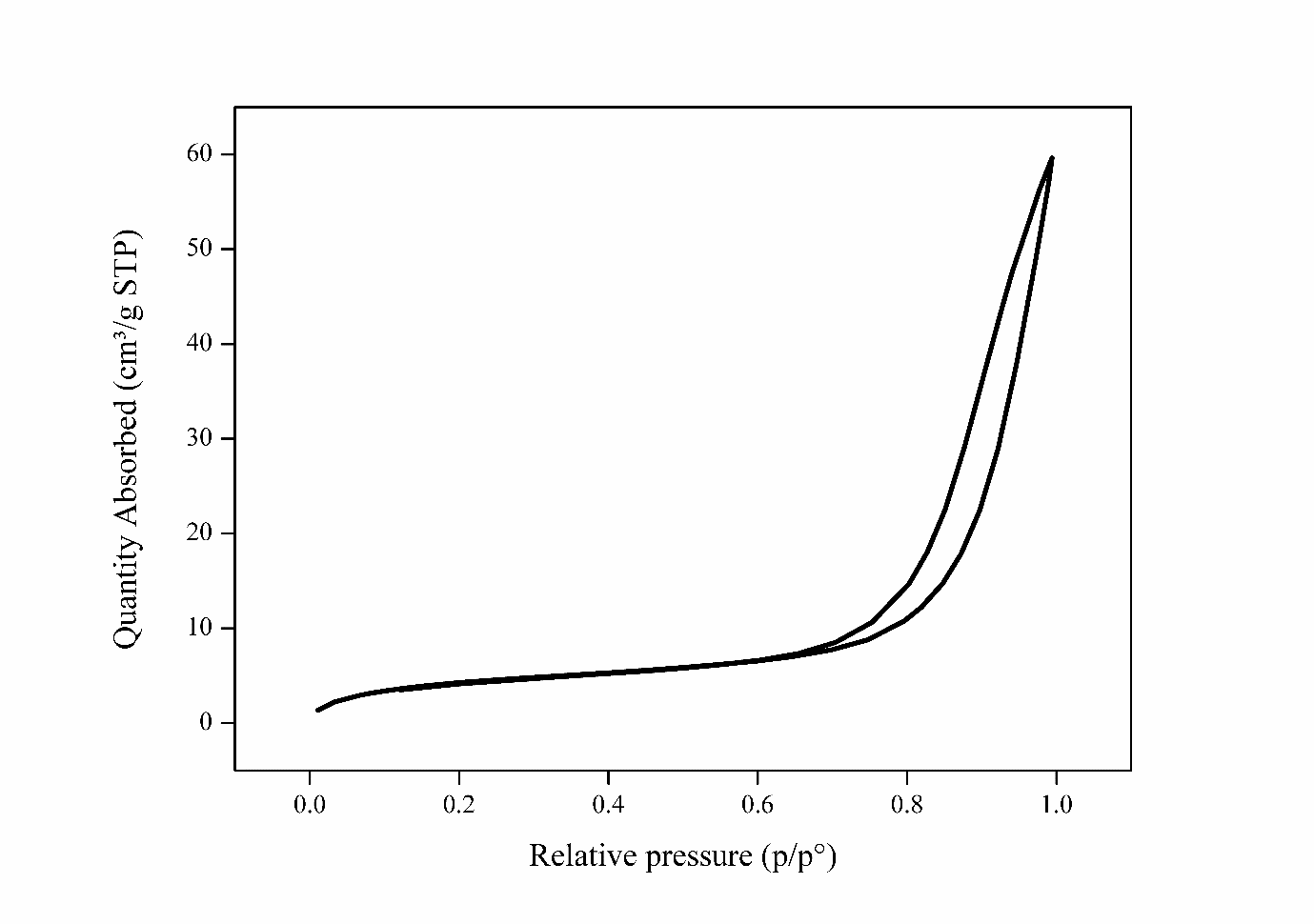


**Figure S3.** BET profile of MSp-TDI-βCD

**Figure S4.** pH_zpc_ for MSp-TDI-βCD
